# A Novel qNMR Application for the Quantification of Vegetable Oils Used as Adulterants in Essential Oils

**DOI:** 10.3390/molecules26185439

**Published:** 2021-09-07

**Authors:** Eleonora Truzzi, Lucia Marchetti, Stefania Benvenuti, Valeria Righi, Maria Cecilia Rossi, Vito Gallo, Davide Bertelli

**Affiliations:** 1 Department of Life Sciences, University of Modena and Reggio Emilia, Via G. Campi 103, 41125 Modena, Italy; eleonora.truzzi@unimore.it (E.T.); lucia.marchetti@unimore.it (L.M.); stefania.benvenuti@unimore.it (S.B.); 2 Doctorate School in Clinical and Experimental Medicine (CEM), University of Modena and Reggio Emilia, 41125 Modena, Italy; 3 Department of Life Quality Studies, Campus of Rimini, University of Bologna, Corso d’Augusto 237, 47921 Rimini, Italy; valeria.righi2@unibo.it; 4 Centro Interdipartimentale Grandi Strumenti, University of Modena and Reggio Emilia, Via G. Campi 213/A, 41125 Modena, Italy; mariacecilia.rossi@unimore.it; 5 Department DICATECh, Politecnico di Bari, Via Orabona 4, 70125 Bari, Italy; vito.gallo@poliba.it

**Keywords:** qNMR, counterfeits, quality control, seed oils, PULCON, triglycerides

## Abstract

Essential oils (EOs) are more and more frequently adulterated due to their wide usage and large profit, for this reason accurate and precise authentication techniques are essential. This work aims at the application of qNMR as a versatile tool for the quantification of vegetable oils potentially usable as adulterants or diluents in EOs. This approach is based on the quantification of both ^1^H and ^13^C glycerol backbone signals, which are actually present in each vegetable oil containing triglycerides. For the validation, binary mixtures of rosemary EO and corn oil (0.8–50%) were prepared. To verify the general feasibility of this technique, other different mixtures including lavender, citronella, orange and peanut, almond, sunflower, and soy seed oils were analyzed. The results showed that the efficacy of this approach does not depend on the specific combination of EO and vegetable oil, ensuring its versatility. The method was able to determine the adulterant, with a mean accuracy of 91.81 and 89.77% for calculations made on ^1^H and ^13^C spectra, respectively. The high precision and accuracy here observed, make ^1^H-qNMR competitive with other well-established techniques. Considering the current importance of quality control of EOs to avoid fraudulent practices, this work can be considered pioneering and promising.

## 1. Introduction

Essential oils (EOs) are complex mixtures of lipophilic volatile substances produced by several aromatic plants as secondary metabolites. EOs are mainly composed by mono- and sesquiterpenes which might be oxygenated or non-oxygenated hydrocarbons. Mono- and sesquiterpenes are usually classified on the basis of the carbon skeleton (cyclic or acyclic) and oxidation status (alcohol, ether, aldehyde, ketone or ester) [1]. The high number of existing terpenoids arises from the huge diversity of the terpene synthases and from the ability of plant enzymes to produce more than one metabolite [2]. As a consequence, depending on the species and varieties of the plants, a great variety of EOs with different compositions are produced by the plant kingdom. EOs display several properties, such as fungicide, bactericide, antioxidant, and insecticide, and since the 13th century, they were being produced by pharmacies and their pharmacological activities were depicted in pharmacopoeias. Nowadays, EOs have proven application in cosmetic, pharmaceutic, and agri-food industries and the scientific interest in these products is more and more increasing due to an interest enhancement in ‘green’ consumerism [3]. In fact, EOs were extensively studied in the last years as natural inhibitors or bio-preservatives in food systems. The application of EOs for food shelf-life extension is mainly due to their antimicrobial and antioxidant activities [4]. Moreover, a wide number of studies have been focusing on EOs as a source for biopesticide, since interesting activities on crop against phytopathogenic fungi, bacteria, and weeds were observed [5,6]. The majority of EOs are generally recognized as safe and for this reason they are commonly used as flavorings in food industries [7].

The great properties of EOs are raising the European and global demand of these valuable products and, consequently, the growing market prompted the adulteration and frauds of EOs. Indeed, the growing demand for EOs has intensified adulteration practices of these valuable products. The counterfeiting of pure EOs can be achieved without declaring by the addition of cheaper synthetic material, cheaper EOs from other natural sources or vegetable oils, with increased profit being the principal incentive [8,9,10]. Indeed, selling essential oils in blend with other components is permissible as long as it is declared on the label by the manufacturer. The counterfeit of these products is an important issue which may reduce consumer confidence and ultimately affect health. Thus, the fast detection and the monitoring of the quantity of adulterating or diluting compounds for industrial purposes is essential but the conventional analytical methods, such as chromatography, are time-consuming and in some cases, useless. For example, gas chromatography (GC), the reference technique for EO analysis [11], is not suitable to ascertain the presence of vegetable oils. As a matter of fact, triglycerides, not being volatile, would be retained in the injection site without reaching the detector.

In this context, other analytical techniques were proposed for this purpose, such as infrared (IR) and nuclear magnetic resonance (NMR) spectroscopy [12,13,14,15]. While IR spectroscopy lacks in identifying molecules sharing a similar structure due to signal overlapping, in the case of NMR different compounds can be discriminated. Indeed, minor differences in the nuclei electron cloud can be finely recorded by NMR spectra. Moreover, since the intensity of spectral signal is only influenced by the number of nuclei in the sample, only one run is necessary for the concomitant identification and quantification (quantitative NMR, qNMR) of many different compounds. These advantages confer the applicability of NMR in food chemistry for quality control of different complex matrices, such as milk, juices, herbal infusions, honey, and wine [16,17,18,19,20]. In our previous work, the application of ^13^C-NMR was attempted for the identification of the vegetable oil used as adulterant in the mixture with EOs. This approach demonstrated to successfully recognize the adulterant in all cases, regardless of the kind of EO employed in the analysis [21]. Therefore, as a continuation of the previous work, a versatile method was set up for the quantification of vegetable adulterants in EOs. This qNMR method might be implemented not only for detecting foreign compounds, but also to verify the real percentage of vegetable oil when added in the mixture, ensuring the amount is consistent with the information reported on the label. For this purpose, a mixture of *Rosmarinus officinalis* (rosemary) EO and corn oil as adulterants was used as a model for the development and further validation of the qNMR method. Afterwards, other EOs, i.e., *Lavandula angustifolia* (lavender), *Cymbopogon nardus* (citronella), and *Citrus sinensis* (orange) were selected for their recurring use in pharmaceutical, cosmetics, and agri-food fields [6,22,23,24,25,26] and randomly blended with corn, peanut, almond, sunflower, and soy seed oils to evaluate the applicability of this method. The choice of vegetable oils was made on a probabilistic basis, considering that they are cheap, easily available, and commonly used since they do not alter the physico-chemical and organoleptic properties of EOs. To the best of our knowledge, no studies aimed to apply qNMR for the development of a general method for vegetable adulterant quantification and quality assessment of EOs, so far. Indeed, the innovative aspect of this work relies not in the novelty of the method, but rather in the application of the well-established qNMR method on EOs.

## 2. Results and Discussion

**Figure 1:**
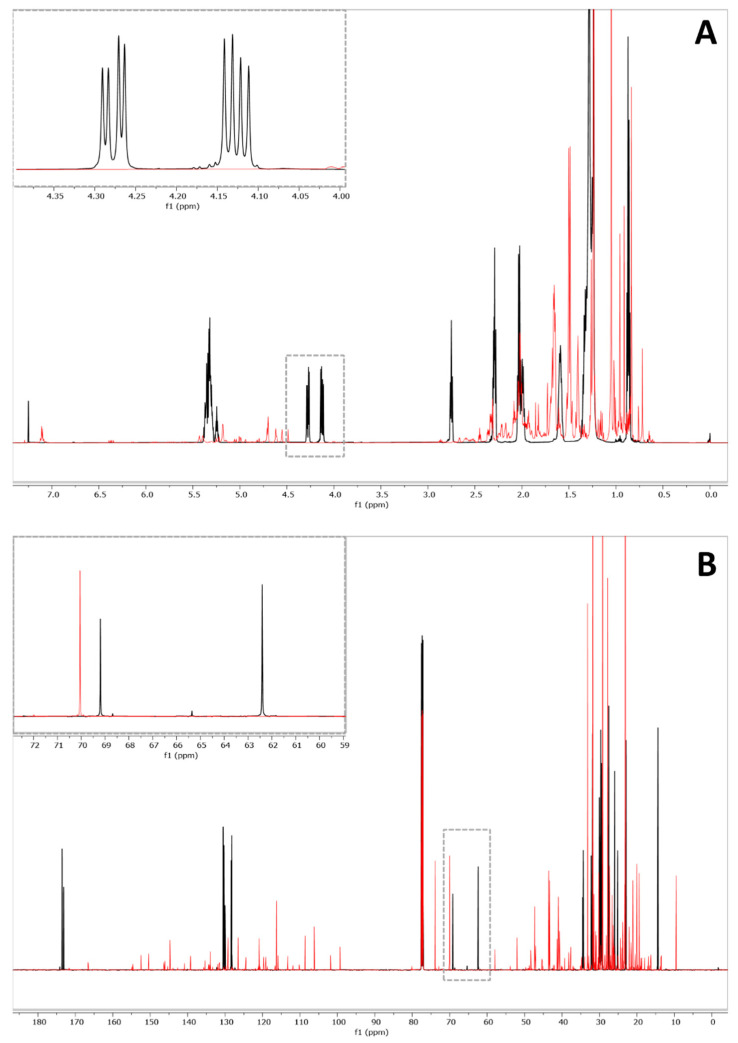
^1^H (**A**) and ^13^C-NMR (**B**) spectra of corn oil (black) and rosemary (red). Enlargements on glycerol backbone signals.

In our previous work, an efficient method for the detection and identification of vegetable oils in mixtures with EOs was developed and validated. The recognizing method was based on the creation of specific chemical fingerprints for the most common vegetable oils, which can be employed as adulterants [21]. Here, the actual quantification of the adulterant content was attempted by employing qNMR both on proton and carbon spectra. The qNMR method makes use of glycerol backbone signals, which are actually present in each vegetable oil containing triglycerides. The quantitative NMR approach was preferred over the construction of calibration curves due to its easy applicability and fast achievement of the result. Indeed, the classic method for quantification with calibration curves requires the preparation of several dilutions of the vegetable oil, increasing both the validation time and the risk of errors. In this work, qNMR with an external reference for the quantification of analytes was employed. The method is based on the PULse Length based CONcentration determination (PULCON) approach. The PULCON methodology correlates the absolute intensity of the signals originating from two different one-dimensional NMR-spectra by the principle of reciprocity [27] through calculating a concentration conversion factor (CCF). The CCF can eventually compensate the signal intensity according to changes in acquisition parameters, such as spectral scan number, receiver gain, pulse length, and tip angle. A particular feature of this method is the avoidance of peak overlapping, between the standard and compound to be quantified. First of all, the quantification method was tested and validated on one combination of EO and vegetable oil (rosemary/corn). Then, the general applicability of this approach was verified on other EO-vegetable oil blends. The ^1^H and ^13^C-NMR spectra of corn oil and rosemary EO were acquired. Regarding the first, in ^1^H-NMR spectra, the signals can be grouped in well-defined regions. Starting from higher frequencies, olefinic protons were detected at 5.39–5.24 ppm, glycerol backbone protons at 5.24, 4.28, and 4.12 ppm, and allylic protons at 2.8–0.80 ppm (Figure 1A). Regarding the ^13^C-NMR spectra, the signals at 174–172 ppm, 133–127 ppm, and 36–13 ppm were identified as carbonyl, unsaturated, and aliphatic carbon atoms, respectively, in accordance with the literature [28] (Figure 1B). The glycerol signals were detected at 62.4 and 69.2 ppm (Figure 1B). The most representative signals of the vegetable oils, which are common to all the vegetable oils considered in the study, were fully described in our previous work [21].

As displayed in Figure 1, the selected rosemary EO sample was unadulterated, without any trace of triglycerides. Furthermore, glycerol backbone signals were isolated, and no peak overlapping was observed. For this reason, binary mixtures of rosemary EO and corn were prepared and employed for method development.

In ^1^H-NMR spectra of the binary mixtures, the glycerol resonances at 4.12 and 4.28 ppm, corresponding both to 2H in position sn 1′ and sn 3′, respectively, (Figure 1A) were integrated as a single signal and accounted for 4 protons. Regarding the ^13^C-NMR spectra, the glycerol signals at 62.4 and 69.2 ppm, corresponding to the two carbons in sn 1′/3′ and one carbon in sn 2′, respectively, were used for quantitation. All these signals were well resolved and exhibited acceptable relaxation times (T_1_). The measured T_1_s were indeed less than 1 s for both protons and carbons, allowing short pulse delays and reasonable times of analysis. For this reason, 5 and 10 s were set as delay times for hydrogen and carbon, respectively, thus ensuring the complete nuclei relaxation. Considering that PULCON allows the employment of any compound as an external reference as long as the acquisition parameters are the same, the choice of using menadione was actually based on its high solubility in chloroform. Moreover, if necessary, menadione could also be used as an internal standard since its signals are not superimposed with those of essential oils and vegetable oils. Since the PULCON method provides the concentration of the molecule in analysis as a molarity, the molar mass of the vegetable oil is required to get the concentration of the adulterant in percentage *w/w*. Since vegetable oils are complex mixtures of triglycerides with different fatty acid ratios, the only possible way to convert the molarity to the *w/w* percentage in the mixture with EOs is to consider the mean molar mass of triglycerides of each vegetable oil. Moreover, since this method aims to a comprehensive analysis of vegetable oils, and the exact composition of the adulterant is obviously unknown, this approximation was necessary. The mean weighted molar mass of triglycerides was calculated considering the mean composition in fatty acids of corn oil reported in the literature [29,30,31]. An average molar mass equal to 872.33 g/mol was used as a conversion factor. For this reason, notwithstanding the PULCON approach, which is highly reproducible, a validation step was necessary since the quantitative calculation is derived from an estimation of the molar mass. The method was validated in terms of inter- and intra-day precision, and the LOD and LOQ values were calculated. Data are reported in Table 1.

The analysis was demonstrated to be adequately precise with RSD lower than 15%, which is the maximum admissible value according to the acceptance criteria of repeatability for the analytical methods. Considering the values of LOD and LOQ, this analytical approach was shown to be sufficiently sensitive for the determination of adulterant oil in EOs. Indeed, the fraudulent addition of counterfeiting would not be profitable at lower percentages than LOQ values.

For the method validation, 14 binary mixtures of rosemary/corn ranging from 0.8 to 50% were prepared. The quantification results on proton and carbon spectra of these binary mixtures are displayed in Table 2. The ^13^C-NMR results were calculated on the mean of the molarity obtained from the two different carbon signals.

The results highlighted that the qNMR approach underestimated the percentage of adulterant oil in the range of 50–1.56% *w/w*. The same tendency was observed for glycerol quantification both in proton and carbon spectra, suggesting that the percent relative error might be due to an instrumental inaccuracy or to an inexact estimation of the average molar mass of corn oil. ^1^H-qNMR demonstrated a higher sensitivity than ^13^C-qNMR, since at a small concentration of adulterant the glycerol intensity was lower than the LOD or LOQ values. This evidence is in accordance with the low natural ^13^C isotopic abundance (~1.1%).

To verify the feasibility of this approach tested here for the first time, further 18 binary mixtures as an external validation sample set were prepared. These mixtures included some of the most appreciated EOs by consumers, i.e., lavender, citronella, and orange. The random percentages of adulterants in the range of 0.8–50% *w/w*, including peanut, almond, sunflower, and soy seed oils, were used to get the highest significant distribution of mixtures to be analyzed. 

**Figure 2:**
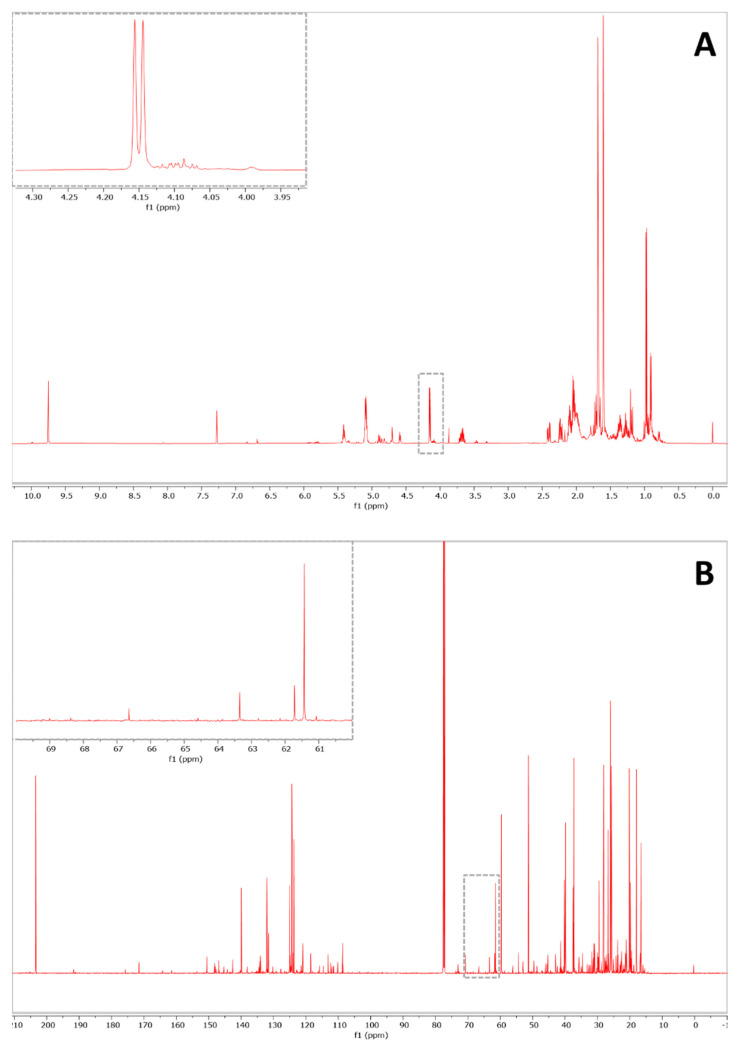
^1^H-NMR and ^13^C-NMR spectra of citronella EO. Enlargements on the typical chemical shifts of glycerol backbone signals.

Prior to the preparation of the binary mixtures, the purity of each EO was assessed by examining the ^1^H and ^13^C-NMR spectra. The glycerol backbone signals were not detected in all the spectra, confirming the absence of triglycerides in the samples. Furthermore, potential signal interferences were evaluated in the region of glycerol signal chemical shifts. In the case of lavender and orange, no potentially interfering typical resonance was detected at glycerol frequencies (4.12–4.28 ppm and 62.4–69.2 ppm for proton and carbon spectrum, respectively). On the contrary, in citronella EO, a doublet was observed in ^1^H-NMR spectrum at 4.15 ppm (Figure 2).

This peak was assigned to the monoterpene geraniol (H-1), which is one of the main components of the EO [32]. Thus, the quantification of the adulterant in ^1^H-NMR spectra of citronella in blends was attempted by integrating only the signals at 4.28 ppm (2H). The determination of the adulterant was performed using the mean molar masses reported in Table 3, calculated on the basis of the composition found in the scientific literature [29,30,31,33]. Quantitative results are reported in Table 4.

The results showed that the efficacy of this approach does not depend on the combination of EO and vegetable oil, but rather the general applicability of this method is confirmed by the variety of the tested mixtures. This evidence supports the wide range of usability of this technique. In the case of citronella EO, the presence of an interfering signal in the region of glycerol did not undermine the outcome of the analysis. Specifically, this overlapping issue was easily overcome by integrating half of the multiplet, originated by the two pairs of glycerol protons.

To the best of our knowledge, existing chemometric tools deal with classifying EO samples by their chemical profiles rather than authenticity control and contaminants detection. However, the application of spectroscopic techniques especially NMR, ATR-FTIR, and GC coupled with chemometrics are an emerging trend in EOs quality assessment [13,34]. Several authors have previously reported the various advantages of IR spectroscopy, including the cost-efficiency, the reduced time of analysis, and the easy spectral data interpretation. On the other hand, the necessity of using calibration curves and the limitations due to the underlying probabilistic approach, make it less convenient [14,35,36]. The potentialities of NMR spectroscopy in quali-quantitative analysis of oils and lipids in general, was formerly disclosed by Lankhorst and Chang [37]. Through the monitoring of typical glycerol backbone chemical shifts and its determination in samples, important information on the authenticity of EOs and on the chemical composition of adulterant can be achieved. This simple approach can make the determination of vegetable oils in EOs much easier. In comparison with analytical techniques involving chemometrics, which usually require a huge number of samples to build a rugged model, this qNMR method is readily feasible. Indeed, a very limited sample preparation and no spectral data pre-treatment or expensive standard compounds are required. The high precision and accuracy here observed, make ^1^H-qNMR competitive with other well-established techniques. Moreover, in the case of carbon, reliable results were obtained. In this context, even if ^13^C-qNMR has some limitations such as poor sensitivity and carbon nuclei long relaxation time, the wider spectral size allows accurate integrations without peak deconvolutions.

The validated approach was then tested on commercial EOs, which resulted as adulterated in our previous work [21]. Among the 20 commercial samples of EOs purchased on the internet, Cymbopogon martinii, Thymbra capitata, Mentha arvensis, and Origanum vulgare EOs were counterfeit with corn, sunflower, soybean, and corn oils, respectively (Table 5). The results achieved on proton and carbon spectra were in good agreement, and highlighted the frequent practice of this kind of fraud. Since these EOs are among the most commonly employed in the food industry, the high percentage of adulteration found in the market remarks the extreme importance of such analytical method for quality control.

## 3. Materials and Methods

### 3.1. Materials

Commercial samples of corn, peanut, almond, sunflower, and soy seed oils were provided from a local marketplace. *Cymbopogon nardus* (citronella), *Lavandula angustifolia* (lavender), and *Rosmarinus officinalis* CT cineole (rosemary) EOs were obtained by Erbamea (Perugia, Italy), while *Citrus sinensis* (orange) EO was kindly donated by Herboris Orientis Dacor (Milan, Italy). Chloroform-*d* (CDCl_3_, 99.8% atom % D), 3-(trimethylsilyl)propionic--2,2,3,3-*d_4_* acid sodium salt (TSP) for internal referencing and menadione as an external reference compound were purchased from Sigma-Aldrich (Milan, Italy). Moreover, 20 commercial samples of EOs were purchased from online shops: *Lavandula angustifolia*, *Thymus vulgaris* (2 samples), *Mentha arvensis* (2 samples), *Cymbopogon martinii*, *Thymbra capitata*, *Eucalyptus globulus*, *Mentha piperita* (2 samples), *Origanum vulgare* (2 samples), *Juniperus communis*, *Ocimum basilicum*, *Citrus limon* (2 samples), *Syzygium aromaticum*, *Chamaemelum nobile*, *Salvia officinalis*, and *Rosmarinus officinalis*.

### 3.2. Sample Preparation

Rosemary, lavender, citronella, and orange EOs were mixed with corn, peanut, almond, sunflower, and soy seed oils in order to obtain binary mixtures at different concentrations ranging from 0.8 to 50% *w/w* of adulterant.

To prepare the NMR tubes, 50 μL of each mixture were diluted with 550 μL of CDCl_3_ (0.03% TMS) directly into a WILMAD^®^ NMR tube, 5 mm, Ultra-Imperial grade, 7 in. L, 528-PP (Sigma-Aldrich, Milan, Italy). The preparation of the tubes was performed by considering the exact weight of the mixture and solvent, in order to easily get the final concentration (*w/w*).

### 3.3. NMR Spectroscopy and Spectra Acquisition Procedures

All the analyses were carried out on a Bruker FT-NMR Avance III HD 600 MHz spectrometer (Bruker Biospin GmbH Rheinstetten, Karlsruhe, Germany). All the experiments were performed at 300 K and non-spinning. The assignments of vegetable oils’ signals were carried out using standard compounds and by comparison of ^1^H-NMR and ^13^C-NMR spectra with literature data in our previous work [21].

^1^H-NMR experiments were acquired using the Bruker sequence “zg30”; the acquisition parameters were as follows: Time domain (number of data points), 131072 K; dummy scans, 2; number of scans, 32; acquisition time, 4.96 s; delay time, 5 s; pulse width, 13 μs; spectral width, 22 ppm; digitization mode, baseopt. The total acquisition time was 5 min and 20 s. ^13^C-qNMR quantification experiments were performed using a 1D inverse gated decoupling sequence to avoid NOE during relaxation. The experiments were acquired using the Bruker sequence “zgpg_pisp_f2.fas” and the acquisition parameters were consequently modified and set as follows: Time domain (number of data points), 65,536 K; dummy scans, 0; number of scans, 256; acquisition time, 0.98 s; delay time, 10 s; spectral width, 220.87 ppm; digitization mode, baseopt. To use this sequence as inverse gated, the proton decoupling power (PLW13) during the recycling delay and experiment time was set to 0 db. The total acquisition time was 47 min. After the sample was inserted into the probe, at least 5 min were waited to achieve the thermal equilibrium. Subsequently, the magnetic field was locked, the probe head was tuned and matched, and finally the sample was shimmed. All these procedures were automatically executed in order to assure the highest reproducibility. For ^1^H-qNMR, the correct 90° pulse was calibrated for each sample with the “pulsecal” Bruker AU program and the receiver gain was set.

### 3.4. Quantitative NMR analysis

Menadione was selected as an external reference compound. The standard solution in CDCl_3_ (9.525 mM) was prepared immediately before the analysis and the spectra were acquired with the same experimental parameters listed above, with an exception for delay times which were set at 30 and 20 s for ^1^H-NMR and ^13^C-NMR, respectively. The ^1^H-NMR signals of menadione used for the quantification were: Singlet at 2.2 ppm (corresponding to the three protons of the methyl group in C_2_), singlet at 6.9 ppm (corresponding to the proton in C_3_), and doublets at 7.8 and 8.2 ppm (corresponding to the two pairs of equivalent aromatic protons). The ^13^C-NMR signal of menadione was the C1′ at 16.8 ppm corresponding to methyl moiety. T_1_ was measured for the signals of menadione and the compounds to be quantified in order to select the correct delay time for the quantification studies. The delay time was set to five times the biggest T_1_. For the ^1^H T1 determination, the Bruker Sequence “T1IR” was used, and the parameters were as follows: A list of 10 increasing delay times (from 10 ms to 30 s); delay time, 30 s; number of scans, 1; total acquisition time, 6 min and 54 s. For the ^13^C T1 determination, the Bruker Sequence “T1irig” was applied, and the parameters were: A list of eight increasing delay times (from 0.1 ms to 100 s); delay time, 150 s; number of scans, 8; total acquisition time, 90 min.

For the quantification, only peaks with a sufficient signal to noise ratio should be used and an exponential window function, with a line broadening (lb) equal to 0.3, should be used for spectra transformation. Prior to the peak integrations, each spectrum was calibrated according to the TMS signal and then an automatic zero order phase and a base-line correction were applied. The quantification of the target compounds was achieved using the Concentration Conversion Factor (CCF) method, implemented in Mnova^®^ 14.1.2 software (Mestrelab Research, S.L., Santiago de Compostela, Spain). The Mnova tool requires a multiplet analysis for the integration. Therefore, after the initial spectra processing, a manual multiplet analysis was carried out, and the peak area of the signals belonging to the triglycerides were compared to the area of signals generated by the menadione standard solution.

The used method correlates with the absolute intensities of two different spectra, since the area of the NMR peak is proportional to the number of nuclei generating this signal under specified conditions [38]. Given the fact that the proportionality relies on pulse excitation, the delay time, broad-band decoupling, and constant acquisition parameters are necessary [39,40]. For the quantification, the peak area of signals belonging to the triglycerides were manually integrated and compared to the area of those generated by the menadione standard solution.

### 3.5. Method Validation

For the method validation, a single combination of EO/adulterant was selected. Precision (intra- and inter-day) was assessed by analyzing binary mixtures of rosemary EO and corn at three different concentrations (low, medium, high) five times for three different days. The results are expressed as average percent relative standard deviations (RSD%). To be on the safe side, the LOQ value is conventionally set at the concentration corresponding to S/N equal to 100, since this ensures the lowest RSD value for the repeated analyses. However, by measuring the lowest adulterant concentration mixtures several times, an acceptable precision was obtained up to signals presenting the S/N ratio equal to 10. For this reason, LOD was determined as the intensity for which the S/N ratio equals 3, conversely LOQ was calculated by considering the area of a signal with a S/N equal to 10. The values of LOD and LOQ are reported as percentages (*w/w*) of the adulterant contained in 50 mL of the NMR tube (final volume equal to 0.6 mL).

## 4. Conclusions

Given that EOs are more and more frequently adulterated due to their wide usage and large profit, accurate and precise authentication techniques are even more essential. The method was able to determine the adulterant percentage, with a mean accuracy value of 91.81 and 89.77% for calculations made on ^1^H and ^13^C spectra, respectively. The accuracy of the quantification was not correlated to the specific EO in the mixture, suggesting the wide feasibility of the method. As a matter of fact, this approach can be potentially applied to all types of EOs, disregarding their composition. This result can be considered successful since the conversion of the molarity was based on an estimated value of molar mass. Indeed, the vegetable oil composition may considerably vary due to abiotic factors, such as temperature, soil salinity, sun exposition, and water supply [41]. Considering the complex composition of EOs and the very limited number of studies employing NMR for the quality control of these valuable products, this tool which improves and finalizes the previous work based on recognition of the adulterant oil, can be considered in our opinion both pioneering and promising.

## Figures and Tables

**Table 1:** Inter- and intra-day precision results, and the LOD and LOQ values were calculated on corn glycerol backbone signals. The precision results are expressed as percent relative standard deviations (RSD%), LOD and LOQ are expressed as percentages (% *w/w*).

Experiment	Signal	Number of Nuclei	Inter-Day RSD% (*n* = 3)	Intra-Day RSD% (*n* = 5)	LOD	LOQ
^1^H-NMR	CH_2_-COOR sn 1′ 3′ (4.12–4.28 ppm)	4	3.21	2.10	0.02%	0.07%
^13^C-NMR	CH_2_-COOR sn 1′ 3′ (62.4 ppm)	2	7.03	6.95	0.64%	2.12%
^13^C-NMR	CH_2_-COOR sn 2′ (69.2 ppm)	1	1.08	2.91	0.82%	2.72%

The nuclei involved in the experiment are underlined in the condensed molecular formula.

**Table 2:** ^1^H and ^13^C qNMR results of rosemary EO—corn oil binary mixtures expressed as adulterant percentage *w/w*.

Expected (% *w/w*)	Measured (% *w/w*)			
	^1^H	Accuracy %	^13^C	Accuracy %
50.30	46.20	91.85	46.90	93.24
50.28	48.11	95.68	46.28	92.04
25.39	21.96	86.49	21.83	85.98
25.36	21.48	84.70	20.26	79.89
13.01	11.83	90.93	11.16	85.78
12.50	11.76	94.08	11.44	91.52
6.37	5.81	91.21	5.93	93.09
6.16	5.87	95.29	5.57	90.42
3.2	2.79	87.19	2.48	77.50
3.11	2.65	85.21	2.51	80.71
1.56	1.32	84.62	<LOQ	-
1.56	1.50	96.15	<LOQ	-
0.79	0.84	106.33	<LOD	-
0.77	0.84	109.09	<LOD	-

**Table 3:** Calculated mean weighted molar masses of vegetable oils included in this study.

Vegetable Oil	Triglycerides Molar Mass (g/mol)
Corn	872.33
Peanut	882.09
Almond	878.32
Sunflower	887.04
Soybean	872.17

**Table 4:** Quantitative results expressed as adulterant percentage (*w/w*) and deviation from the real content of adulterant oil.

EO/Adulterant	Expected (% *w/w*)	Measured Adulterant (% *w/w*)			
		^1^H	Accuracy %	^13^C	Accuracy %
Lavender/almond	51.10	44.24	86.58	45.78	89.59
Lavender/sunflower	40.03	39.81	99.45	38.09	95.15
Lavender/peanut	36.16	34.32	94.91	31.75	87.80
Lavender/almond	13.54	12.81	94.61	12.13	89.59
Lavender/soy	3.29	3.18	96.66	3.03	92.10
Lavender/peanut	0.90	0.76	84.56	<LOQ	-
Citronella/soy	53.74	48.18	89.65	48.43	90.12
Citronella/peanut	40.00	40.37	100.93	39.40	98.50
Citronella/sunflower	36.61	29.19	79.73	29.57	80.77
Citronella/soy	16.89	15.31	90.65	15.26	90.35
Citronella/sunflower	5.49	4.62	84.15	4.25	77.47
Citronella/almond	3.93	3.50	89.11	3.22	81.98
Orange/sunflower	51.37	52.06	101.34	50.98	99.24
Orange/soy	50.83	46.33	91.15	47.50	93.45
Orange/almond	48.39	44.14	91.22	44.08	91.09
Orange/peanut	6.09	5.90	96.88	6.21	101.97
Orange/almond	3.72	3.02	81.18	2.87	77.15
Orange/sunflower	0.95	0.95	99.89	<LOQ	-

**Table 5:** Quantitative results of vegetable oils expressed as adulterant percentage (*w/w*) found in commercial samples of EOs.

EO/Adulterant	Measured Adulterant (% *w/w*)	
	^1^H	^13^C
*Cymbopogon martinii* (Roxb.)/corn	32.80	33.11
*Thymus capitatus* (Cav.)/sunflower	41.67	43. 34
*Mentha arvensis* (L.)/soy	10.27	10.29
*Origanum vulgare* (L.)/corn	17.81	17.46

## Data Availability

The data presented in this study are available on request from the corresponding author.

## Sample Availability

All samples are available from the authors.
